# An Improved African Vulture Optimization Algorithm for Dual-Resource Constrained Multi-Objective Flexible Job Shop Scheduling Problems

**DOI:** 10.3390/s23010090

**Published:** 2022-12-22

**Authors:** Zhou He, Biao Tang, Fei Luan

**Affiliations:** 1 School of Electrical and Control Engineering, Shaanxi University of Science & Technology, Xi’an 710021, China; 2 College of Mechanical and Electrical Engineering, Shaanxi University of Science & Technology, Xi’an 710021, China

**Keywords:** improved African vulture algorithm, dual-resource constrained flexible job shop scheduling problem, population initialization, memory bank, neighborhood search operation

## Abstract

According to the characteristics of flexible job shop scheduling problems, a dual-resource constrained flexible job shop scheduling problem (DRCFJSP) model with machine and worker constraints is constructed such that the makespan and total delay are minimized. An improved African vulture optimization algorithm (IAVOA) is developed to solve the presented problem. A three-segment representation is proposed to code the problem, including the operation sequence, machine allocation, and worker selection. In addition, the African vulture optimization algorithm (AVOA) is improved in three aspects: First, in order to enhance the quality of the initial population, three types of rules are employed in population initialization. Second, a memory bank is constructed to retain the optimal individuals in each iteration to increase the calculation precision. Finally, a neighborhood search operation is designed for individuals with certain conditions such that the makespan and total delay are further optimized. The simulation results indicate that the qualities of the solutions obtained by the developed approach are superior to those of the existing approaches.

## 1. Introduction

With the intensification of economic globalization, the production cycle of products has been reduced, requiring enterprises to establish and use manufacturing systems in an agile manner. In the current production system, scheduling is essential for increasing labor efficiency and productivity, as well as enhancing an organization’s ability to compete. At present, many studies focused on the analysis of the problem of flexible job shop scheduling (FJSP) can be found. For example, Gao et al. [1] have proposed an improved artificial bee colony algorithm for FJSP with fuzzy processing time. Li et al. [2] have developed a hybrid artificial bee colony algorithm based on Tabu search for emergencies in FJSP and a clustered grouping roulette method to better initialize the population. Caldeira et al. [3] have presented an improved Jaya algorithm, using a local search method, an effective initialization mechanism, and an acceptance criterion to improve the quality of the FJSP solution. Zhang et al. [4] have recently shown that a genetic algorithm (GA) based on a variable neighborhood search can be used to address the NP-hard property of FJSP, providing a strong search capability and diversity. To solve the FJSP, Chen et al. [5] have used a self-learning genetic algorithm that automatically adjusts the vital parameters of the GA through reinforcement learning. An improved GA based on hybrid initialized populations, manual cross-over methods, and adaptive weighting mechanisms has been proposed by Zhang et al. [6], in order to optimize multi-objective FJSP. Ding et al. [7] have investigated FJSP, and obtained a high-quality scheduling scheme by improving the encoding–decoding scheme, the inter-particle communication mechanism, and the substitution rules for the candidate operation machines in a particle swarm optimization algorithm. Li et al. [8] have developed a scheduling solution based on the Monte Carlo tree search algorithm for the dynamic flexible job shop scheduling problem, in order to minimize the makespan. Different Petri-net based heuristic scheduling methods were used to obtain optimal or near-optimal schedules for FJSP [9,10,11,12,13]. The FJSP has been solved by Caldeira et al. [14] using a discrete multi-objective Jaya algorithm, taking the makespan, total machine workload, and workload of essential machines as performance metrics. There have been many studies on FJSP that consider machine constraints, and many excellent results have been achieved. However, FJSP in the actual manufacturing process is not only constrained by machines, but also by workers. Therefore, it is evident that the traditional job shop scheduling problem (JSP) of machine constraints and FJSP of machine constraints do not meet the requirements of actual production. As a result, the dual-resource flexible job shop scheduling problem (DRCFJSP) with machine and worker constraints has been proposed. The gap between theoretical and practical scheduling problems can be reduced by considering the DRCFJSP. However, as the DRCFJSP is an extension of JSP and FJSP, it still faces significant challenges, such as inheriting the NP-hard features of JSP and FJSP.

At present, there are few relevant studies on DRCFJSP. Cao et al. [15] have contraposed the NP-hard nature of DRCFJSP and, based on the information processing mechanism of innate immunity in biological science, a novel immune genetic algorithm combining the immune algorithm and GA was proposed. Li et al. [16] have presented a branching population genetic metaheuristic algorithm, in order to minimize the completion time and cost of DRCFJSP. The algorithm is a GA-based scheduling method that accumulates and transmits the evolutionary experience of parental chromosomes by introducing pheromones into the branching population. Zheng et al. [17] have designed a knowledge-guided fruit fly optimization algorithm based on a novel coding method to solve the DRCFJSP including a processing time minimization criterion. Zhang et al. [18] have studied the DRCFJSP, and designed a hybrid discrete particle swarm optimization algorithm. Zhang et al. [19] have used a quantum genetic algorithm (QGA) for the DRCFJSP, in order to improve the efficiency of the QGA solution through quantum coding, niche techniques to initialize the population, adaptive rotation angles, and quantum mutation strategies. Tan et al. [20] have proposed a fatigue-based DRCFJSP to alleviate worker fatigue and improve productivity through the joint scheduling of machines and workers.

With the dawn of Industry 4.0 [21], DRCFJSP has become a hot topic of study. As mentioned above, many algorithms have been employed to address the DRCFJSP, in order to obtain scheduling solutions with better overall performance. The African vulture optimization algorithm (AVOA) [22] is a novel metaheuristic optimization algorithm that simulates the foraging and navigation behavior of vultures. Its effectiveness and superiority have been demonstrated through its application to various engineering design problems. Nevertheless, there have been few investigations on AVOA in scheduling problems, even though it has potential advantages in solving scheduling problems.

To adapt to the complicated and volatile external environment of enterprises, in this paper, we construct a makespan and total delay target model, and improve the AVOA (IAVOA) through various improvement strategies to shorten the production cycle time of products, thus increasing the competitive advantage of enterprises. In addition, we also compare and analyze IAVOA against a number of widely used multi-objective algorithms, in order to evaluate relevant performance metrics.

The remainder of this paper is structured as follows: Section 2 presents the specific details of the problem model. The algorithm adopted to solve the model is detailed in Section 3. The improvement strategy and the solution process of IAVOA are provided in Section 4. The simulation experiment is introduced in Section 5. Finally, Section 6 concludes the work.

## 2. The Multi-Objective DRCFJSP Model

### 2.1. Description of the Problem

In the following, we provide a description of the DRCFJSP. Suppose that there are w workers in a workshop using m machines to process n jobs. The set of jobs is denoted as $J=\{J_{1},J_{2},\ldots ,J_{n}\}$, the set of machines as M={1,2,…,m}, and the set of workers as W={1,2,…,w}. A job $J_{i}$ has $n_{i}$ operations waiting for processing, and there is a constraint on the sequence between operations. Each operation $O_{i,j}$ requires $m^{\prime }$ candidate machines, and each machine $M_{P}$ has $w^{\prime }$ candidate workers. Therefore, there are cases where the processing time $PT_{i,j,p,k},k\in W$ of the (same or different) machines processing operation $O_{i,j}$ differs. The objective of scheduling is to arrange the processing of n jobs reasonably. Therefore, the three tasks of operation sequence, machine allocation, and worker selection comprise the DRCFJSP.

In this paper, we construct a DRCFJSP model to optimize the makespan and total delay. To describe the DRCFJSP model, relevant parameters are defined in Table 1.

To obtain a better scheduling solution for the DRCFJSP, the following assumptions are made: (1) there is no hierarchy of importance for various jobs; (2) there is a precedence constraint between various operations of the same job, but not between operations of different jobs; (3) each worker is only able to operate one machine at a time to complete one operation; (4) each operation can only be processed on one machine; (5) each operation process cannot be interrupted; (6) the processing time for all jobs can be zero; and (7) the transfer time of workers on different machines is the same as that of jobs on different machines.

### 2.2. Objective Function

1. Makespan

(1) $$f_{1}=\min(Max(C_{i}),i\in \{0,1,2\ldots ,n\}),$$

2. Total delay

The total delay objective function has been proposed by Jun [23]. The formula to calculate the total delay $f_{2}$ is as follows:

(2) $$f_{2}=\min(\sum_{i=1}^{n}ww_{i}\cdot T_{i}),$$

(3) $$T_{i}=Max(0,C_{i}-d_{i}),$$

such that

(4) $$\begin{array}{l} C_{i,j}\geq C_{i,(j-1)}+BT_{i,j,p,k}+\max(H_{i,(j-1),p^{\prime },i,j,p}\cdot TT_{i,(j-1),p^{\prime },k,i,j,p,k},H_{i,(j-1),k^{\prime },i,j,k}\cdot TT_{i,(j-1),p,k^{\prime },i,j,p,k})\\ i\in \{1,2,\ldots ,n\},j\in \{1,2,\ldots ,n_{i}\},(p,p^{\prime })\in M,(k,k^{\prime })\in W \end{array},$$

(5) $$C_{i^{\prime },j^{\prime }}+(1-Y_{i,j,p,i^{\prime },j^{\prime },p})\cdot L\geq C_{i,j}+\sum_{k=1}^{w}BT_{i,j,p,k}\cdot X_{i,j,p,k}(i,i^{\prime })\in \{1,2,\ldots ,n\},j\in \{1,2,\ldots ,n_{i}\},j^{\prime }\in \{1,2,\ldots ,n_{i^{‘}}\},(p,p^{\prime })\in M,(k,k^{\prime })\in W,$$

(6) $$\begin{array}{l} C_{i^{\prime },j^{\prime }}+(1-Z_{i,j,k,i^{\prime },j^{\prime },k})\cdot L\geq C_{i,j}+\sum_{p=1}^{m}BT_{i,j,p,k}\cdot X_{i,j,p,k}+H_{i^{\prime },j^{\prime },k,i,j,k}\cdot TT_{i^{\prime },j^{\prime },p^{\prime },k^{\prime },i,j,p,k}\\ (i,i^{\prime })\in \{1,2,\ldots ,n\},j\in \{1,2,\ldots ,n_{i}\},j^{\prime }\in \{1,2,\ldots ,n_{i^{‘}}\},(p,p^{\prime })\in M,k\in W \end{array},$$

(7) $$\sum_{p=1}^{m}\sum_{k=1}^{w}X_{i,j,p,k}=1,i\in \{1,2,\ldots ,n\},j\in \{1,2,\ldots ,n_{i}\},$$

As shown in Equation (4), there are restrictions on the order in which the job can be processed. Equation (5) indicates that, with each machine running at the same time, only one operation can be processed. Equation (6) implies that there is only one job that each worker can process at the same time. Finally, Equation (7) means that an operation can only be performed once.

## 3. African Vulture Optimization Algorithm

The African Vulture Optimization Algorithm (AVOA) [22] is a recently proposed metaheuristic algorithm that is divided into an exploration phase and an exploitation phase, mimicking the foraging and navigation behavior of African vultures. The exploitation phase can be divided further into a co-operative phase and a competitive phase. The AVOA determines the phase of the algorithm mainly based on the hunger level F of the vulture. If $F<r_{1}$, AVOA enters the exploration phase. If $F>r_{2}$, AVOA moves into the co-operative phase. If the above two conditions are not satisfied, AVOA enters the competition phase. The formula for calculating a vulture’s hunger level F is as follows:

(8) $$t=h\times \left(\sin^{\omega }\left(\frac{\pi }{2}\times \frac{t}{\max t}\right)+\cos\left(\frac{\pi }{2}\times \frac{t_{i}}{\max t}\right)-1\right),$$

(9) $$F=\left(2\times rand_{1}+1\right)\times z\times \left(1-\frac{t_{i}}{\max t}\right)+t,$$

where $t_{i}$ defines the present number of iterations, maxt defines the total number of iterations, z is a number drawn at random from the range [−1,1], h is a number chosen at random from the range [−2,2], $rand_{1}$ is a number selected at random from the range [0,1], and ω is a constant value (ω=1).

(1) Exploration phase: Vultures are superb foragers and have outstanding vision in the wild, so they can spot dying animals. However, vultures may have great difficulty in finding food, and need to check different random regions to find food. In AVOA, random regions are represented by two distinct position update formulas, and the parameter $P_{1}$ is used to select the areas searched by vultures. The random number rand is used to determine the position update formula to obtain new individuals in the exploration phase. If $rand\geq P_{1}$, the position update formula Equation (10) is used; otherwise, the position update Equation (13) is used:
  (10) $$P\left(i+1\right)=R\left(i\right)-D\left(i\right)\times F,$$
  (11) $$D\left(i\right)=\left|X\times R\left(i\right)-P\left(i\right)\right|,$$
  (12) $$X=2\times rand_{1},$$
  (13) $$P\left(i+1\right)=R\left(i\right)-F+rand_{2}\times \left(\left(ub-lb\right)\times rand_{3}+lb\right),$$
  where $P\left(i\right)$ is the position vector of the vultures to be updated; $P\left(i+1\right)$ is the new vulture position vector; $R\left(i\right)$ indicates either an optimal or sub-optimal vulture; $rand_{1}$, $rand_{2}$, and $rand_{3}$ are random numbers between [0,1]; lb and ub represent the upper and lower bounds of the variables; and X defines how vultures move at random to defend their prey from other vultures.
(2) Co-operative phase: When the vultures are hungry, they will move collectively in search of food. The food source can be represented by two different position update formulas. The random number rand is used to determine the position update formula for the new individual in this phase. If $rand\geq P_{2}$, Equation (14) is applied. Otherwise, Equation (17) is applied.
  (14) $$P\left(i+1\right)=D\left(i\right)\times \left(F+rand_{4}\right)-d\left(t\right),$$
  (15) $$d\left(t\right)=R\left(i\right)-P\left(i\right),$$
  (16) $$\begin{array}{l} S_{1}=R\left(i\right)\times \left(\frac{rand_{5}\times P\left(i\right)}{2\pi }\right)\times \cos\left(P\left(i\right)\right)\\ S_{2}=R\left(i\right)\times \left(\frac{rand_{6}\times P\left(i\right)}{2\pi }\right)\times \sin\left(P\left(i\right)\right) \end{array},$$
  (17) $$P\left(i+1\right)=R\left(i\right)-\left(S_{1}+S_{2}\right),$$
  where $rand_{4}$, $rand_{5}$, and $rand_{6}$ are a random numbers between [0,1].
(3) Competitive phase: Serious disputes over food availability can arise when a large group of vultures gather at a single food source. Food sources can be represented by two different position update formulas. The random number rand is used to determine the position update formula followed by the new individual in the competitive phase. If $rand\geq P_{3}$, Equation (19) is applied; otherwise, Equation (20) is applied.
  (18) $$\begin{array}{l} A_{1}=BV_{1}\left(t\right)-\frac{BV_{1}\left(t\right)\times P\left(i\right)}{BV_{1}\left(t\right)-P{\left(i\right)}^{2}}\times F\\ A_{2}=BV_{2}\left(t\right)-\frac{BV_{2}\times P\left(i\right)}{BV_{2}-P{\left(i\right)}^{2}}\times F \end{array},$$
  (19) $$P\left(i+1\right)=\frac{A_{1}+A_{2}}{2},$$
  (20) $$P\left(i+1\right)=R\left(i\right)-\left|d\left(t\right)\right|\times F\times LF\left(d\right),$$
  (21) $$LF\left(x\right)=0.01\times \frac{u\times \sigma }{{\left|v\right|}^{\frac{1}{\beta }}},\sigma ={\left(\frac{\Gamma \left(1+\beta \right)\times \sin\left(\frac{\pi \beta }{2}\right)}{\Gamma \left(1+\beta 2\right)\times \beta \times 2\left(\frac{\beta -1}{2}\right)}\right)}^{\frac{1}{\beta }},$$
  where $BV_{1}\left(t\right)$ is the optimal vulture in the current iteration, $BV_{2}\left(t\right)$ is the sub-optimal vulture in the current iteration, $d\left(t\right)$ is the distance between $BV_{1}\left(t\right)$ and $BV_{2}\left(t\right)$, LF refers to Levy flight, d refers to the dimension of the problem, u and v are both arbitrary numbers between [0,1], and the default value for β is fixed at 1.5.

## 4. Improved African Vulture Optimization Algorithm (IAVOA)

### 4.1. Initialization of the Population

According to previous studies, a high-quality initial population is helpful in increasing the accuracy of such an algorithm, as well as balancing its exploration and development capacities. Therefore, we designed three rules to generate initial population individuals, as follows:

1. The shortest processing time principle: Determine the sequence of operations and the selection of machines in a random manner. For the allocation of workers, select the workers with the shortest processing time;
2. The machine–worker integration principle: Determine the sequence of operations in a random manner, fix the workers assigned to each machine at random, randomly determine the selection allocation of machines, and determine the selection of workers according to the selection of machines;
3. The randomization principle: Determine the sequence of operations, the selection of machines, and the distribution of workers in a random manner.

The overall processing time of the jobs can be decreased by using the shortest processing time principle. On the contrast, the machine–worker integration principle can reduce the transfer time caused by worker transfer. These two methods have a greater probability of producing better individuals than random methods; however, to maintain population diversity and prevent the algorithm from converging too early, the proportion of individuals generated by the shortest processing time principle and machine–worker integration principle should be low. Thus, 20% of the individuals are generated by the shortest processing time principle, 10% of the individuals are generated by the machine–worker integration principle, and finally, 70% of the individuals are generated by the randomization principle.

### 4.2. Solution Representation

The DRCFJSP studied in this paper can be divided into three sub-problems: sequencing of operations, allocation of machines, and selection of workers. Therefore, we designed a three-segment encoding scheme—specifically, including a sequence code for operations OC, an allocation code for machines MC, and a selection code for workers WC. The size of each segment is the sum of the operations included in the scheduling problem. The size of the chromosome is $\sum_{i=1}^{n}n_{i}$, where $n_{i}$ reflects the number of operations in job i. Each gene on a bit in OC corresponds to a job number, and different occurrences of the same job number correspond to different operations of the task. In MC, each gene represents that a qualified machine $M_{k}$ is selected from the machine set M for processing operation $O_{ij}$. For WC, each gene represents a worker who uses the machine. The three-segment encoding method is conducive to optimizing the processing time and other information in the decoding process, and reducing the complexity of algorithm calculation.

**Figure 1:**
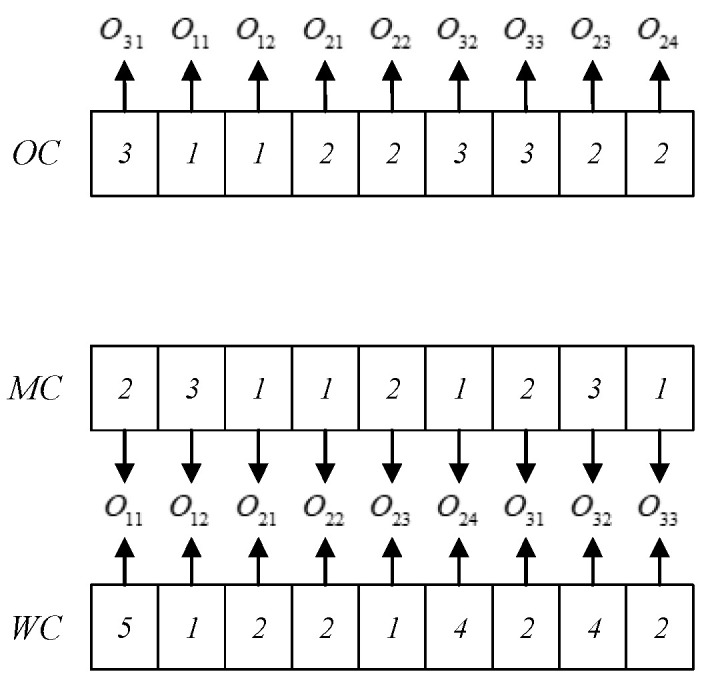
Three-segment encoding scheme.

Figure 1 shows an example of the three-segment coding. This example includes three jobs $\left\{J_{1},J_{2},J_{3}\right\}$, three machines $\left\{m_{1},m_{2},m_{3}\right\}$, and five workers $\left\{w_{1},w_{2},w_{3},w_{4},w_{5}\right\}$. The workers $\left\{w_{2},w_{4}\right\}$ can utilize machine $m_{1}$, workers $\left\{w_{1},w_{2},w_{5}\right\}$ can utilize machine $m_{2}$, and workers $\left\{w_{1},w_{3},w_{4}\right\}$ can utilize machine $m_{3}$. From left to right, the OC is scanned. The first "3" illustrates the first operation $O_{31}$ of job $J_{3}$, the first "1" illustrates the first operation $O_{11}$ of job $J_{1}$, and the second "3" illustrates the second operation $O_{32}$ of job $J_{3}$. Therefore, the processing sequence of the corresponding operation in Figure 1 is $O_{31}\to O_{11}\to O_{12}\to O_{21}\to O_{22}\to O_{32}\to O_{33}\to O_{23}\to O_{24}$. In MC, the first “2” represents machine $m_{2}$ processing operation $O_{11}$, the first “1” represents machine $m_{1}$ processing operation $O_{12}$, and the second “1” represents machine $m_{1}$ processing operation $O_{21}$. In WC, the number “5” indicates that worker $w_{5}$ uses machine $m_{2}$ to process operation $O_{11}$ (i.e., $\left\{w_{5},m_{2},O_{11}\right\}$), and the number “1” indicates that worker $w_{1}$ uses machine $m_{3}$ to process operation $O_{12}$. Based on the above, the specific processing information of this example can be expressed as follows:

$$\left\{w_{2},m_{2},O_{31}\},\{w_{5},m_{2},O_{11}\},\{w_{1},m_{3},O_{12}\},\{w_{2},m_{1},O_{21}\},\{w_{2},m_{1},O_{22}\},\{w_{4},m_{3},O_{32}\},\{w_{2},m_{1},O_{33}\},\{w_{1},m_{2},O_{23}\}\{w_{4},m_{1},O_{24}\right\}$$

### 4.3. Individual Position Vector

**Figure 2:**
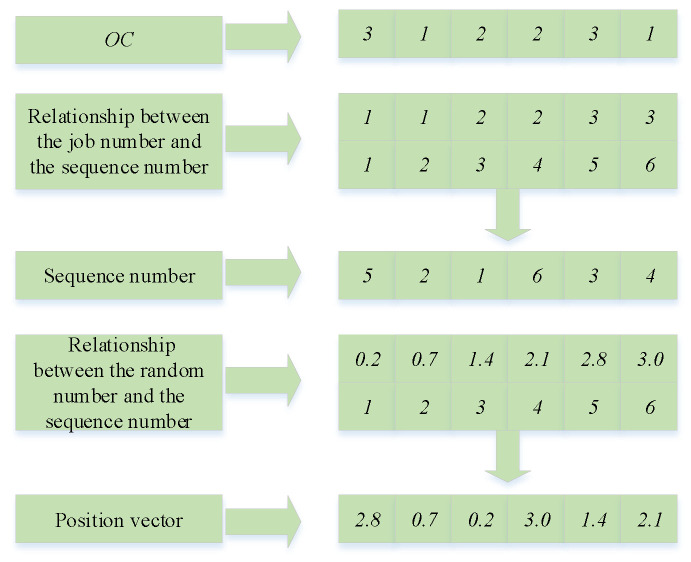
Individual position vector.

The initial position vector is determined by the OC. The position vector is composed of random numbers in the range [−n,n], with length of $\sum_{i=1}^{n}n_{i}$. Figure 2 shows an example of generating a position vector. In Figure 2, there are three jobs $\left\{J_{1},J_{2},J_{3}\right\}$ to be processed, where each job includes two operations. It can be seen in Figure 2 that the OC of individual $P_{1}$ is 3→1→2→2→3→1. Obtaining the sequence number according to the relation between the sequence number and the job number gives 5→2→1→6→3→4. Obtaining the position vector using the relation between the random number and the sequence number gives 2.8→0.7→0.2→3.0→1.4→2.1. 

### 4.4. Fitness

When solving DRCFJSP by IAVOA, each individual has different degrees of fitness. Therefore, it is crucial that we develop a way to assess an individual’s fitness value. In evaluating individual fitness, the single objective optimization problem only requires comparing the value of the objective function. However, for problems regarding multi-objective optimization, due to the mutual constraints between objectives, it is necessary to measure the target value of each individual comprehensively. Common methods for evaluating comprehensive individual performance are SPEA2’s *k*-nearest neighbors method, NSGA-Ⅱ’s Pareto frontier method, and the weight method, among others. To better assess the fitness of individuals and obtain the optimal global solution, we designed a new formula to calculate the individual fitness value, as follows:

(22) $$F=\sum_{l=1}^{s}r_{l}\cdot f_{{}_{l}}^{2},$$

where s is the dimension of the objective function, l is the index of the objective function, f is the objective function, and $r_{l}$ is a number drawn at random from the range [0,1]. In this paper, to prevent the algorithm from entering local optima and maintain high population diversity, the parameter $r_{l}$ is used to control the search direction of the population.

### 4.5. Memory Bank

The memory bank is a simple storage unit, which is used to store the better individuals in the population. The memory bank has a full file value $N_{A}$, which controls the population size. The memory bank judges the fitness of individuals according to certain rules. To improve the overall quality of the next generation of individuals, we designed two rules to select better individuals:

1. Boundary rule: The boundary value $\alpha_{l}$ of the population is calculated by Equation (23), and individuals smaller than the boundary value are stored in the memory bank. The boundary rule can eliminate individuals with large target values, reduce the number of invalid searches in the solution space, and enhance the algorithm’s computational effectiveness.
2. Distance rule: The distance between each individual and the optimal individual is calculated, and individuals with a large distance are deleted from the memory bank. The distance rule can control the algorithm’s search direction, focusing it toward the optimal solution.

The specific steps of the memory bank processing are as follows:

Step 1: Use Equation (23) to calculate the boundary value $\alpha_{l}$ of the population and calculate the Euclidean distance between the individuals and the optimal individual. Individual distances in the population are sorted in ascending order:
  (23) $$\alpha_{l}=\max f_{l}-r\cdot \max f_{l},$$
  where l defines the dimension of the objective function, and r refers to a number in the interval [0,1] (e.g., r=0.35);
Step 2: Individuals less than the boundary value $\alpha_{l}$ of any dimension using the boundary rule are stored in the memory bank;
Step 3: Determine whether the number of individuals in the memory bank has reached $N_{A}$. If the number of individuals l in the memory bank is higher than $N_{A}$, individuals with a large distance, according to the distance rule, are deleted; otherwise, the storage of individuals in the memory bank is completed.

### 4.6. Updating of Individuals

IAOVA uses position vectors to update individuals in the population. First, we use the location update formula of AVOA to update the individual location vector in the memory bank, and then use the individual update mechanism to update the individuals. For this paper, we designed an operation update mechanism, three machine–worker update mechanisms, and a neighborhood search operation. The specific steps by which IAOVA updates individuals are as follows:

Step 1: Determine the optimal vulture individual, the sub-optimal vulture individual, and the individual $P_{i}$;
Step 2: Update the position vector of $P_{i}$ using the position update formula of AVOA;
Step 3: Use the process update mechanism to update the OC of individuals in the memory bank;
Step 4: Determine whether the neighborhood search condition is reached. If the condition is fulfilled, the neighborhood search operation is executed; otherwise, proceed to Step 5;
Step 5: Randomly generate a random number rr in the range [0,1];
Step 6: SUse rr to determine the machine–worker update mechanism. Update MC and WC of individuals in the memory bank.

#### Individual Updating Mechanism

1. Operation update mechanism

The specific steps for updating individual operation sequences are as follows:

Step 1: θ is determined randomly, where θ is a position element of the position vector of individual $P_{i}$;
Step 2: Carry out ascending ranking of element values higher than θ in the position vector of individual $P_{i}$, and record the changed operation numbers and position vector elements;
Step 3: Set the operation number of the optimal vulture or the sub-optimal vulture to zero, which is the same as the operation number recorded in Step 2, and record the non-zero numbers and position vector elements;
Step 4: Record the operation number and position vector in Step 2 as the first half of the sub-generation, and record the operation number and position vector in Step 3 as the second half of the sub-generation.

**Figure 3:**
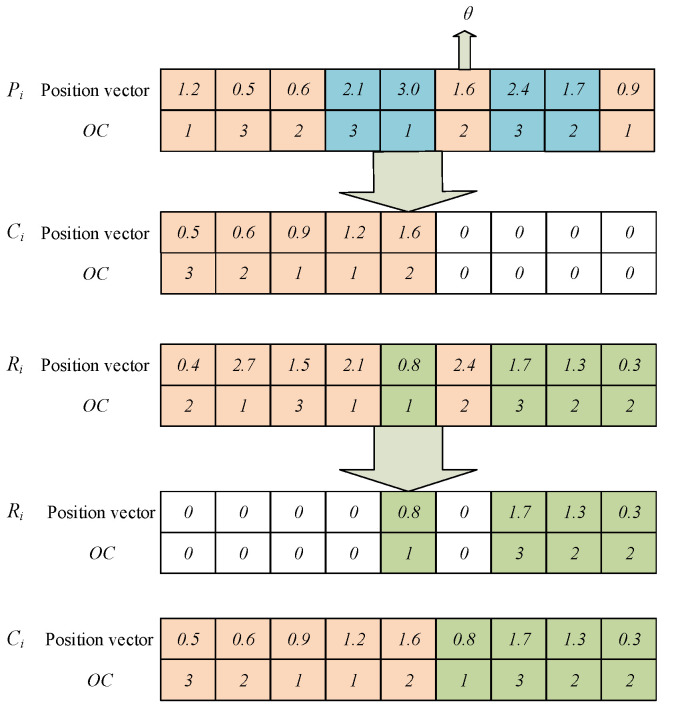
Operation update mechanism of the individual.

The operation update mechanism of the individual is depicted in Figure 3.

2. Machine–worker update mechanism

Based on the relationship between the AVOA solution and the optimal or sub-optimal vulture, and to maintain the diversity of the algorithm, we designed three machine–worker update mechanisms. The specific mechanisms are as follows.

(1) Machine–worker self-updating mechanism

Step 1: Generate two location indices $l_{1}$ and $l_{2}$ on MC in a random way;
Step 2: Determine the processing procedures on the location indices $l_{1}$ and $l_{2}$, and randomly select machines from the machine set to replace the machines at these locations;
Step 3: Randomly select workers to replace workers at these locations.

**Figure 4:**
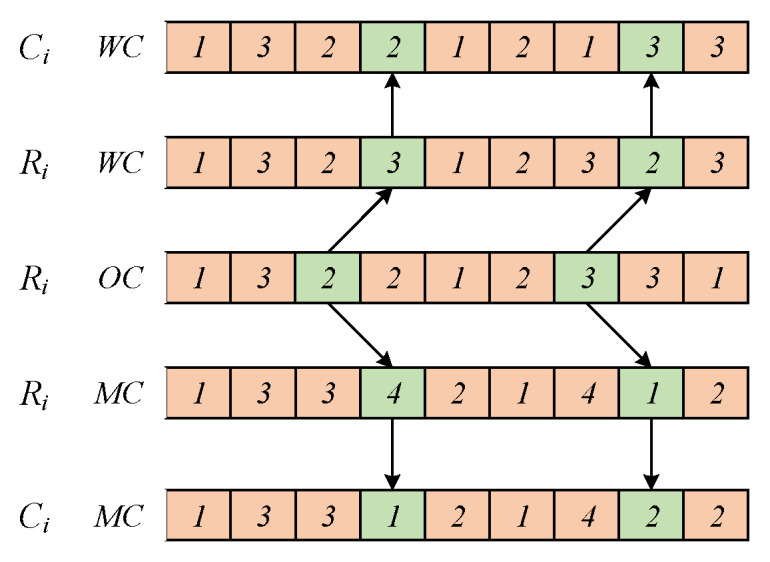
Self-updating process for individual machine–worker.

Figure 4 depicts the machine–worker self-updating process of individuals.

(2) Machine–worker cross-updating mechanism

Step 1: Calculate the exchange digit l using Equation (24):
  (24) $$l=round(u\cdot \sum_{i=1}^{n}n_{i}),$$
  where round is the rounding function, and u=0.225.
Step 2: Randomly generate two location indices $l_{1}$ and $l_{2}$, where $|l_{1}-l_{2}|=l$;
Step 3: The position elements between $l_{1}$ and $l_{2}$ in vulture $R_{i}$ are transferred to the offspring $C_{i}$ in turn, keeping the original location index unchanged;
Step 4: The values of individual $P_{i}$, except for the location elements between the location indices $l_{1}$ and $l_{2}$, are kept unchanged and passed to the child $C_{i}$ in turn.

**Figure 5:**
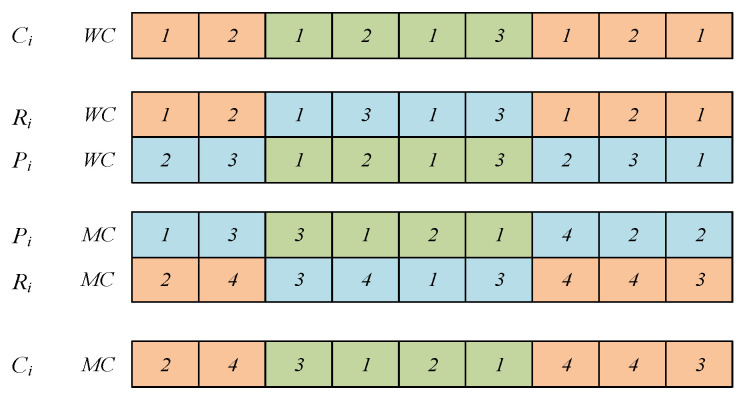
Cross-updating process for individual machine–worker.

Figure 5 depicts the individual machine–worker cross-updating process.

(3) Worker self-updating mechanism

Step 1: Step 1: Randomly generate two location indices $l_{1}$ and $l_{2}$ on WC;
Step 2: Set the location elements in the location indices $l_{1}$ and $l_{2}$ of the individual $P_{i}$ to be updated to the child $C_{i}$;
Step 3: In individual $R_{i}$, except for the location elements between the location indices $l_{1}$ and $l_{2}$, the original location indices are kept unchanged and passed to the child $C_{i}$ in turn.

**Figure 6:**
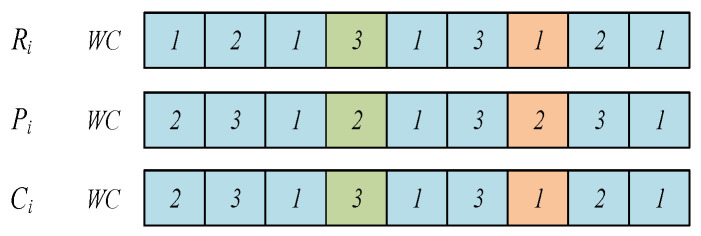
Updating process for individual WC.

The new MC is the MC of vulture $R_{i}$. Figure 6 depicts the WC updating process.

### 4.7. Neighborhood Search Operation

The neighborhood search operation enhances the algorithm’s exploration of the solution space, enhances the quality of the solution for a wide variety of individual groups, and improves the ability of the algorithm to skip local optima. The neighborhood search operation is as follows:

Step 1: Judge whether an individual’s position vector meets the neighborhood search conditions (more than 60% of the elements on the position vector are the same, or all elements on the position vector are boundary values). If the condition is met, Step 2 is performed. Otherwise, the neighborhood search operation is not performed;
Step 2: Determine the allele exchange number $N_{g}$;
Step 3: Generate the location indices $l_{1}$ and $l_{2}$;
Step 4: In OC, the alleles on the location indices $l_{1}$ and $l_{2}$ are exchanged. For the position vector, the elements located at $l_{1}$ and $l_{2}$ are generated randomly;
Step 5: Judge whether the exchange number $N_{g}$ has been met. If the condition is met, new individuals will be generated. Otherwise, Step 2 is executed.

**Figure 7:**
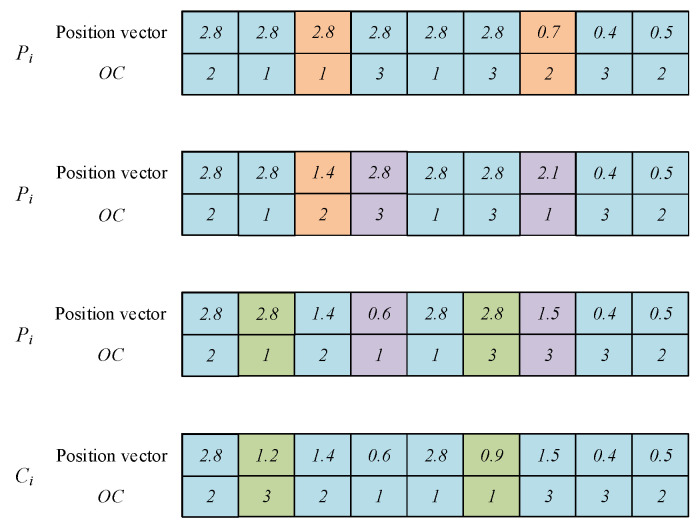
Operation flow of the neighborhood search operation.

The specific operation flow of the neighborhood search operation is shown in Figure 7. Suppose that the allele exchange number $N_{g}$ is 3. Each exchange of alleles does not affect each other. Therefore, there is a certain probability of generating the same location index. For example, in Figure 7, the location index required for the second exchange is the same as that generated the first time.

### 4.8. Framework of the Developed Algorithm

**Figure 8:**
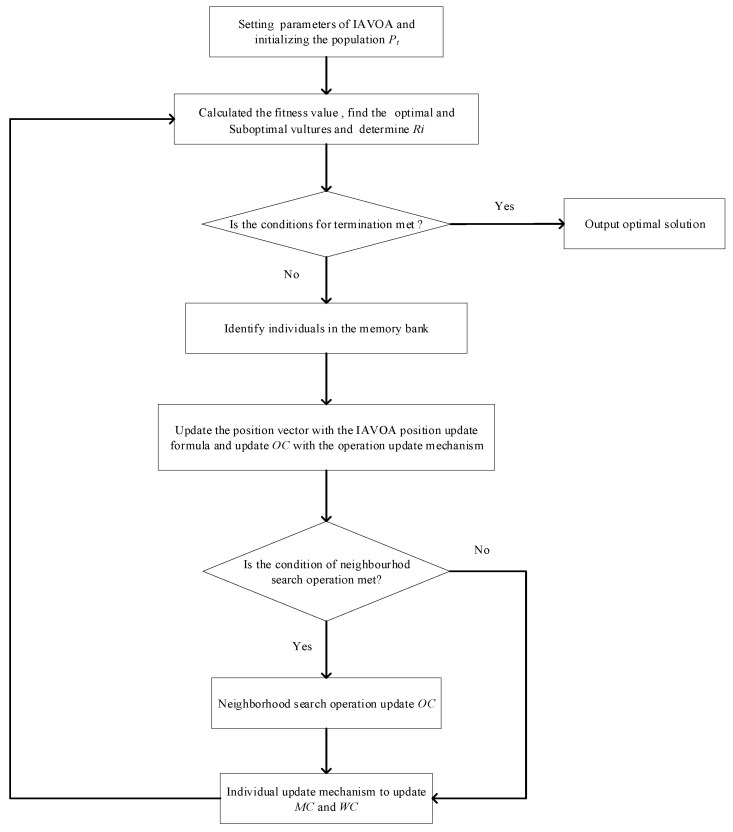
Flow chart of the IAVOA process.

The AVOA is improved by designing the initial population and memory bank, balancing the ability of the AVOA to explore and develop the solution space. The neighborhood search operation is intended to enhance the ability of the algorithm to skip local optima. Figure 8 shows a flow chart indicating how IAVOA is used to solve the DRCFJSP. The specific steps of the solution process are as follows:

Step 1: Set up the parameters;
Step 2: Initialize the population $P_{t}$ by using the strategy for population initialization;
Step 3: Calculate individual fitness values in the population. Select the optimal vulture and the sub-optimal vulture, and determine the vulture $R_{i}$;
Step 4: Judge whether the termination condition has been met. If the condition is fulfilled, Step 9 is executed. Otherwise, Step 5 is executed;
Step 5: Compose the population $P_{t}$ and memory bank $A_{t}$ into a new memory bank $A_{t}^{\prime }$, and calculate the Euclidean distance and boundary value $\alpha_{l}$ between the individuals and the optimal vulture in $A_{t}^{\prime }$;
Step 6: Use the memory bank pruning strategy to prune $A_{t}^{\prime }$ to obtain the memory bank $A_{t+1}$;
Step 7: Update the position vector of $A_{t+1}$ using the position update formula of AVOA, and update the OC of $A_{t+1}$ using the operation update mechanism;
Step 8: Judge whether the neighborhood search operation is required. If the condition is fulfilled, the neighborhood search operation is used to update the individuals. Otherwise, the machine–worker update mechanism updates the individual machine code and the worker code. New individuals join the new population $P_{t+1}$. Then, Step 3 is executed;
Step 9: Output the optimal solution.

## 5. Experimental Simulation and Analysis

We analyze the performance of IAVOA through an experiment. The environment for the experimental analysis was Windows 10, with 4 GB of RAM and an Intel i7 processor, and the programming environment was MATLAB 2021. The experimental analysis included the following.

(1) Setting the parameters of IAVOA;
(2) Assessing the optimal performance of IAVOA;
(3) Examining the performance of IAVOA, compared to that of commonly used multi-objective optimization algorithms.

### 5.1. Evaluation Metrics

To assess the performance of IAVOA, three evaluation metrics are used in this paper: Generational Distance (*GD*) [24], Inverse Generational Distance (*IGD*) [25], and hypervolume (*HV*) [26]. *GD* and *IGD* were used to evaluate the convergence of IAVOA, while *HV* was employed to estimate the diversity of IAVOA. The formulas for these evaluation metrics are as follows:

1. *GD*

(25) $$GD=\frac{\sqrt{\sum_{i=1}^{\left|\Omega \right|}D_{{}_{i}}^{2}\left(\Omega ,\;\;\;\Omega^{*}\right)}}{\left|\Omega \right|},$$

where Ω is the first Pareto front value (PF) obtained by an algorithm, $\Omega^{*}$ is the true PF value, |Ω| is the quantity of elements in the Pareto front obtained from the algorithm’s solution, and $D_{i}(\Omega ,\;\Omega^{*})$ denotes the minimum Euclidean distance that separates the solution in $\Omega^{*}$ from solution i in Ω. A smaller *GD* value indicates better convergence of the algorithm.

2. *IGD*

(26) $$IGD=\frac{\sum_{i=1}^{\left|\Omega^{*}\right|}D_{i}\left(\Omega^{*},\Omega \right)}{\left|\Omega^{*}\right|},$$

where $\left|\Omega^{*}\right|$ is the quantity of elements in the true PF, and $D_{i}(\Omega^{*},\Omega )$ denotes the shortest Euclidean distance that separates the solution in Ω from solution i in $\Omega^{*}$. A smaller value of the *IGD* indicates better convergence of the algorithm.

3. *HV*

(27) $$HV=\delta (\cup_{i=1}^{\left|\Omega \right|}V_{i}),$$

where δ is the Lebesgue measure, and $V_{i}$ denotes the hypercube formed between the reference point and the solution i in PF. A larger *HV* value reflects better diversity of the algorithm.

### 5.2. Test Case

As the DRCFJSP is a relatively new problem, there were no standard cases for testing the algorithm’s performance. Therefore, we applied 24 test cases, DMK01–DMK15 and DDP10–DDP18, based on FMK01–FMK15 and FDP10–FDP18, respectively [20]. The quantity of jobs, operations, machines, and workers; the processing time $BT_{i,j,p,k}$ of the operation; and the transit time $TT_{i,(j-1),p^{\prime },k^{\prime },i,j,p,k}$ of the two machines in the test case were the same as in FMK01–FMK15 and FDP10–FDP18. The delivery period of jobs is related to the tightness factor of the delivery period [27]. Equation (28) describes the delivery period for job i. The weights for the jobs were generated using the weight averaging method. Table 2 shows the scale used in the test case.

(28) $$d_{i}=r_{i}+c\times \sum_{j=1}^{n_{i}}\overset{*}{BT_{i,j,p,k}},$$

where $\overset{*}{BT_{i,j,p,k}}$ indicates the mean processing time for operation j of job i, $r_{i}$ denotes the release time of job i ($r_{i}\in U[0,\sum_{j=1}^{n_{i}}\overset{*}{BT_{i,j,p,k}}/n_{i}]$), and *c* indicates the tightness factor of the delivery period (c=1.2).

### 5.3. Setting of Parameters

**Figure 9:**
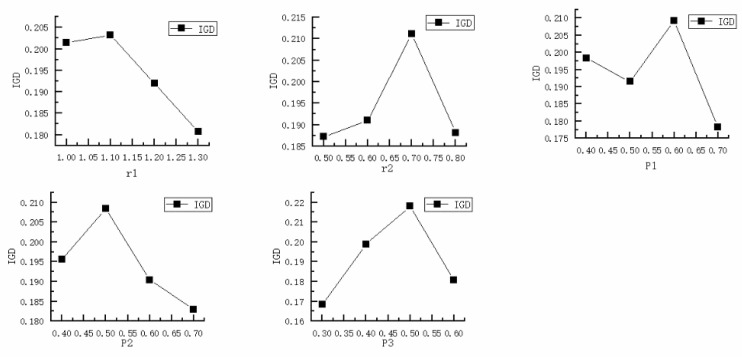
Factor level trend of the parameters.

The performance of IAVOA is significantly impacted by the parameter settings. The key parameters affecting the performance of the IAVOA are $r_{1}$(i.e., $r_{1}\in [1,1.3]$), $r_{2}$(i.e., $r_{2}\in [0.5,0.8]$), $P_{1}$(i.e., $P_{1}\in [0.4,0.7]$), $P_{2}$(i.e., $P_{2}\in [0.4,0.7]$), and $P_{3}$(i.e., $P_{3}\in [0.3,0.6]$), where $r_{1}$ and $r_{2}$ determine the phase of the IAVOA (i.e., exploration, co-operative, or competition), and $P_{1}$, $P_{2}$, and $P_{3}$ primarily determine the location update formula employed by IAVOA at each phase. In this paper, the DMK08 was considered a test case, and the Taguchi method [28] for setting up an orthogonal experiment was applied to determine the best parameter combination strategy. Several reasonable levels for the parameters are given in Table 3, and each group of parameters was run 10 times, where the average value for each *IGD* group was regarded as the response value. Table 4 displays the average response values and the rank of parameters. As shown in Table 4, the parameters with the highest impact rank were $r_{2}$ and $P_{3}$. Figure 9 depicts the factor level trend of the parameters. The optimal combination of parameters for IAVOA was found to be $r_{1}=1.3$, $r_{2}=0.5$, $P_{1}=0.7$, $P_{2}=0.7$, and $P_{3}=0.3$, through experimental analysis.

### 5.4. Performance Analysis of IAVOA

In this subsection, the methods developed in [24,29] are employed as comparison algorithms to assess the performance of IAVOA (i.e., SPEA2 and NSGA-II). Both algorithms have been widely used and their excellent performance in solving engineering problems has been proven. Therefore, they were considered highly reliable for comparison. The differences of the two multi-objective optimization algorithms are as follows: SPEA2 evaluates individual fitness mainly by the *k*-nearest neighbor method, whereas NSGA-II evaluates individual fitness by the non-dominated sorting technique and crowding distance calculation approach. Table 5 shows the parameter setting values for the different algorithms. Zitzler [24] has identified that SPEA2 produces superior solutions around $P_{c}=0.8$ and $P_{m}=0.1$. Therefore, the probability of crossing and mutation for SPEA2 in Table 5 was set as 0.8 and 0.15, respectively. To avoid errors arising from the parameters, the NSGA-II probability of crossing and mutation was also set as 0.8 and 0.15. The range of each parameter was shown in parentheses. For each case, we run the tested algorithm 10 times.

**Figure 10:**
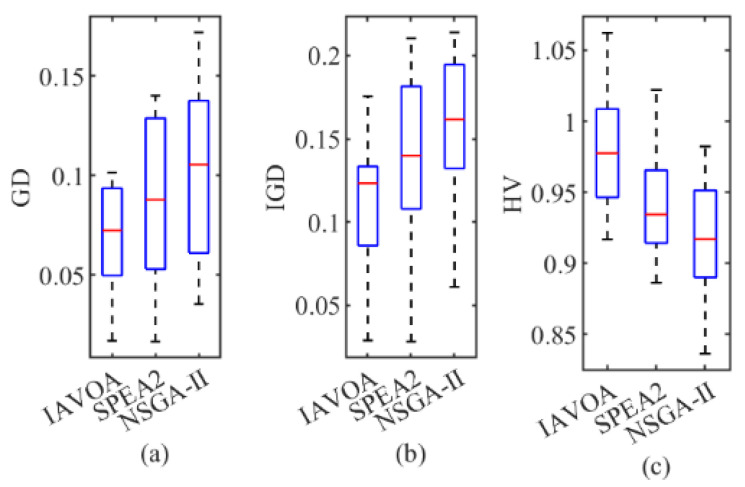
Box plot for the three evaluation metrics: (**a**) *GD*; (**b**) *IGD*; (**c**) *HV*.

The performance metrics of the three algorithms are displayed in Table 6 where the data in bold indicate the optimal values of the performance metrics for each test case. Multiple test cases are detailed in Table 6, where IAVOA’s GD and *IGD* values were better than those of SPEA2 and NSGA-II, demonstrating that IAVOA has better convergence. The data in Table 6 indicated that IAVOA also had better *HV* values than SPEA2 and NSGA-II, except for the cases DMK01, DMK04, and DMK07, which demonstrates that the diversity of IAVOA is generally better than the other two algorithms. Figure 10 shows the box plots for the three evaluation metrics. 

**Figure 11:**
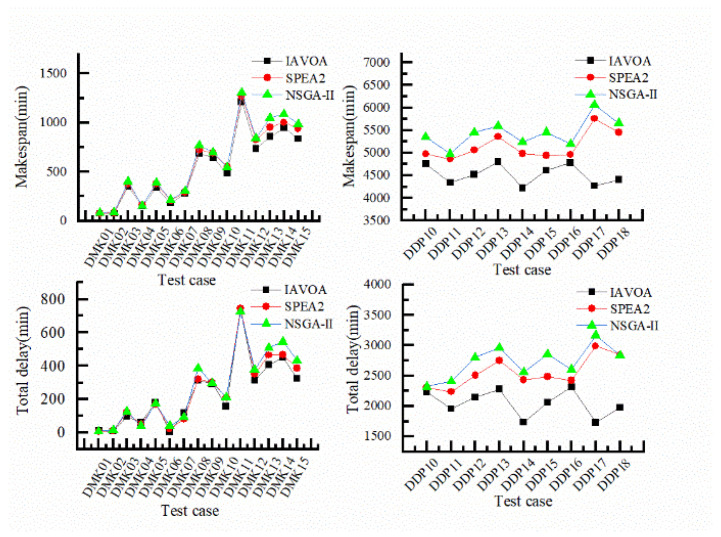
Line chart of the optimization problem values.

Table 7 shows the optimization values for the test cases resolved using IAVOA, SPEA2, and NSGA-II. The data in bold indicate the optimal results for the corresponding cases solved by the algorithms, and the corresponding line chart is shown in Figure 11, which demonstrate that higher quality scheduling solutions were obtained when solving DRCFJSP with IAVOA, compared to the other two algorithms. Figure 11 illustrates the various advantages of IAVOA in solving the large-scale DRCFJSP. Thus, our experiment showed that IAVOA is better suited to the practical FJSP solution than SPEA2 and NSGA-II.

**Figure 12:**
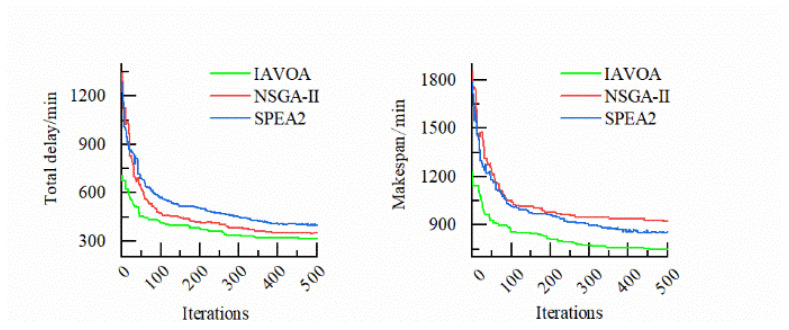
Convergence graph of DMK12.

**Figure 13:**
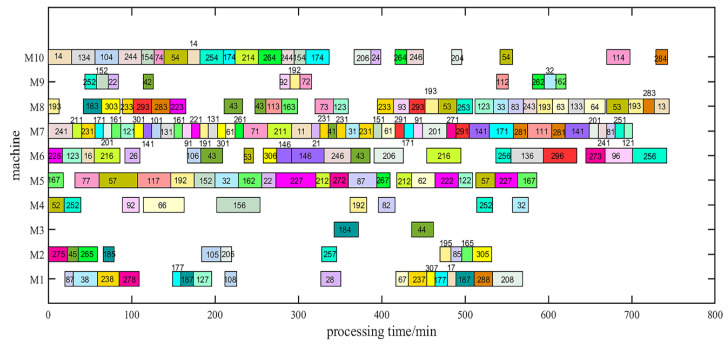
Gantt chart of DMK12.

To clearly determine the performance of IAVOA, the medium-scale DMK12 was selected for performance analysis. Figure 12 presents the convergence graph for DMK12, which shows that IAVOA provides a better solution when the iterations increase. The superior results obtained by IAVOA illustrate the feasibility and effectiveness of the improved strategy. Figure 13 shows the Gantt chart of DMK12 for IAVOA.

## 6. Conclusions

In this paper, we considered the influence of machine and worker constraints on the FJSP, and established the DRCFJSP with makespan and total delay as objective functions. The AVOA, based on the position update formula and position vector, was proposed to solve DRCFJSP. To sustain population diversity and optimize solution quality, the population was initialized in three ways, following the shortest processing time principle, the machine–worker integration principle, and the randomization principle. The memory bank of AVOA was improved to enhance the solution space exploration and exploitation capabilities of the algorithm. A neighborhood search operation was designed to avoid the algorithm falling into local optima, and the Taguchi method was employed to determine the optimal parameters of the algorithm. The test cases were DMK01-DMK10 and DDP10-DDP18, based on FMK01-FMK10 and FDP10-FDP18, respectively. The experimental results demonstrated that the IAVOA can outperform the state-of-the-art SPEA2 and NSGA-II in solving large-scale flexible job shop scheduling problems. In terms of performance metrics, the experiments verified that IAVOA has good convergence and diversity.

For future work, there exist many dynamic problems attached to FJSP, such as job insertion, machine breakdowns, and other emergencies, which deserve further consideration. In this manuscript, the limitation of overtime for workers was not considered. This problem can be solved in future work by increasing the number of workers. Distributed scheduling and reverse scheduling can also be considered as future research directions. In addition, the algorithm needs further improvement, in order to obtain a scheduling solution with better performance.

## Figures and Tables

**Table 1:** Definitions of Parameters.

Symbol	Description
n	Quantity of jobs
m	Quantity of machines
w	Quantity of workers
$n_{i}$	Total number of the job i operations
$O_{i,j}$	The j th operation of job i
$BT_{i,j,p,k}$	The processing time of the j th operation of worker k using machine p to process job i
$TT_{i,(j-1),p^{\prime },k^{\prime },i,j,p,k}$	Transfer time of job i for two adjacent operations on the machines or workers
$C_{i}$	Completion time of job i
$d_{i}$	The delivery date of job i
$T_{i}$	Delay time of job i
$ww_{i}$	Weight coefficient of job i
L	A big real number
$H_{i,(j-1),p^{\prime },i,j,p}$	If two adjacent operations of job i are processed on the different machines, $\;H_{i,(j-1),p^{\prime },i,j,p}=1$; otherwise, $\;H_{i,(j-1),p^{\prime },i,j,p}=0$
$H_{i,(j-1),k^{\prime },i,j,k}$	If two adjacent operations of job i are processed by the different workers, $H_{i,(j-1),p^{\prime },i,j,p}=1$; otherwise, $H_{i,(j-1),p^{\prime },i,j,p}=0$
$X_{i,j,p,k}$	If the operation $O_{i,j}$ is processed on machine p by $w_{k}$ $,\;X_{i,j,p,k}=1$; otherwise, $X_{i,j,p,k}=0$
$Y_{i,j,p,i^{\prime },j^{\prime },p}$	If the sequence of different operations for processing different jobs is processed on machine p $,\;Y_{i,j,p,i^{\prime },j^{\prime },p}=1$; otherwise, $Y_{i,j,p,i^{\prime },j^{\prime },p}=0$
$Z_{i,j,k,i^{\prime },j^{\prime },k}$	If the sequence of different operations for processing different jobs is processed by worker k $,\;Z_{i,j,k,i^{\prime },j^{\prime },k}=1$; otherwise, $Z_{i,j,k,i^{\prime },j^{\prime },k}=0$

**Table 2:** Scale of the test case.

Test Case	Job	Operation	Machine	Worker
DMK01	10	55	6	4
DMK02	10	58	6	4
DMK03	15	150	8	6
DMK04	15	90	8	5
DMK05	15	160	2	3
DMK06	10	150	15	10
DMK07	20	100	5	4
DMK08	20	225	10	8
DMK09	20	240	10	8
DMK10	20	240	15	10
DMK11	30	179	5	4
DMK12	30	193	10	8
DMK13	30	231	10	8
DMK14	30	277	15	12
DMK15	15	293	8	6
DDP10	15	293	8	6
DDP11	15	293	8	6
DDP12	15	293	8	6
DDP13	20	387	10	8
DDP14	20	387	10	8
DDP15	20	387	10	8
DDP16	20	387	10	8
DDP17	20	387	10	8
DDP18	20	387	10	8

**Table 3:** Level of parameters.

Parameter	Level			
	1	2	3	4
$r_{1}$	1	1.1	1.2	1.3
$r_{2}$	0.5	0.6	0.7	0.8
$P_{1}$	0.4	0.5	0.6	0.7
$P_{2}$	0.4	0.5	0.6	0.7
$P_{3}$	0.3	0.4	0.5	0.6

**Table 4:** Response values and rank of parameters.

Level	$r_{1}$	$r_{2}$	$P_{1}$	$P_{2}$	$P_{3}$
1	0.2015	0.1872	0.1984	0.1956	0.1686
2	0.2033	0.1910	0.1915	0.2084	0.1990
3	0.1919	0.2111	0.2092	0.1905	0.2180
4	0.1807	0.1881	0.1783	0.1830	0.1807
Delta	0.0208	0.0239	0.0309	0.0254	0.0373
Rank	5	4	2	3	1

**Table 5:** Parameter setting values of the different algorithm.

Parameter	IAVOA	SPEA2	NSGA-Ⅱ
Population size	100 (100~300)	100 (100~300)	100 (100~300)
Iterations	500 (400~700)	500 (400~700)	500 (400~700)
The probability of crossing $P_{c}$	-	0.8 (0.75~0.85)	0.8 (0.75~0.85)
The probability of mutation $P_{m}$	-	0.15 (0.5~0.2)	0.15 (0.5~0.15)
Memory bank $N_{A}$	100 (100~500)	-	-
Archive	-	100 (100~500)	-
$r_{1}$	1.3(1~1.3)	-	-
$r_{2}$	0.5(0.5~0.8)	-	-
$P_{1}$	0.7(0.4~0.7)	-	-
$P_{2}$	0.7(0.4~0.7)	-	-
$P_{3}$	0.3(0.3~0.6)		

**Table 6:** Performance metric values of the three algorithms.

Test Case	*GD*			*IGD*			*HV*		
	IAVOA	SPEA2	NSGA-Ⅱ	IAVOA	SPEA2	NSGA-Ⅱ	IAVOA	SPEA2	NSGA-Ⅱ
DMK01	0.0168	0.0164	0.0353	0.029	0.0283	0.0611	1.0087	1.0221	0.9701
DMK02	0.0280	0.0564	0.0764	0.0483	0.0976	0.1322	1.0250	0.9612	0.9152
DMK03	0.0943	0.1287	0.1428	0.1334	0.1815	0.2019	0.9861	0.9183	0.8945
DMK04	0.0546	0.0529	0.0477	0.1443	0.1397	0.1258	0.9169	0.9264	0.9513
DMK05	0.0496	0.0703	0.0874	0.0859	0.1218	0.1513	1.0622	1.0187	0.9823
DMK06	0.0901	0.1401	0.1720	0.0901	0.1401	0.172	0.9600	0.8863	0.8365
DMK07	0.0527	0.0483	0.0609	0.1178	0.1079	0.1361	0.9464	0.9656	0.9336
DMK08	0.0911	0.1052	0.1376	0.1289	0.1487	0.1946	0.9689	0.9424	0.8796
DMK09	0.1014	0.1214	0.1235	0.1755	0.2103	0.2139	0.9363	0.8930	0.8901
DMK10	0.0935	0.1369	0.1342	0.1322	0.1936	0.1898	0.9989	0.9143	0.9191
DMK11	0.1065	0.1234	0.1300	0.1506	0.1745	0.1838	1.0003	0.9691	0.9626
DMK12	0.0695	0.1784	0.1120	0.1204	0.1784	0.1939	0.9661	0.8915	0.8663
DMK13	0.1333	0.1867	0.2366	0.1333	0.1867	0.2366	0.9922	0.9195	0.8551
DMK14	0.0600	0.0692	0.0948	0.1586	0.183	0.2507	1.0457	1.0118	0.9167
DMK15	0.0982	0.1558	0.1752	0.1387	0.2188	0.2477	1.0358	0.9197	0.8849
DDP10	0.1420	0.1654	0.2007	0.1942	0.2326	0.2839	0.9642	0.9218	0.8652
DDP11	0.0893	0.1386	0.1502	0.1785	0.2771	0.3003	1.0692	0.9334	0.8996
DDP12	0.0887	0.1256	0.1561	0.1984	0.2809	0.349	0.9352	0.8268	0.7436
DDP13	0.0721	0.1123	0.1265	0.2164	0.2751	0.3098	1.0290	0.8935	0.8470
DDP14	0.1423	0.2449	0.2750	0.2013	0.3464	0.3889	1.0549	0.8540	0.7999
DDP15	0.1182	0.1637	0.2169	0.2046	0.2836	0.3757	1.0887	0.9718	0.8503
DDP16	0.0749	0.0840	0.0977	0.1675	0.1879	0.2184	0.9396	0.9128	0.8722
DDP17	0.0540	0.1584	0.1759	0.0935	0.2744	0.3046	1.0970	0.8454	0.8072
DDP18	0.0930	0.1793	0.1879	0.1611	0.3106	0.3254	1.0563	0.8503	0.8334

**Table 7:** Objective function values for the test cases solved with the three algorithms.

Test Case	Optimal Results of Makespan/min			Optimal Results of Total Delay/min		
	IAVOA	SPEA2	NSGA-Ⅱ	IAVOA	SPEA2	NSGA-Ⅱ
DMK01	75	76	83	11.7687	7.4946	7.9494
DMK02	75	82	91	10.0309	12.1499	17.1734
DMK03	349	379	400	97.4101	121.1134	126.3537
DMK04	150	162	155	61.9927	49.9123	42.0513
DMK05	338	379	390	182.5554	170.4126	174.6927
DMK06	183	205	218	6.4038	23.2212	40.4034
DMK07	280	293	306	118.9131	82.1253	93.6101
DMK08	687	726	769	312.6511	321.0397	386.9322
DMK09	643	690	699	291.3925	301.9603	297.0488
DMK10	485	556	550	158.5083	212.4392	211.066
DMK11	1211	1271	1306	735.8705	744.534	725.7022
DMK12	736	828	842	313.419	351.2002	376.6815
DMK13	858	954	1050	407.6391	465.5957	510.1178
DMK14	947	1001	1091	451.6775	466.4827	545.0523
DMK15	836	938	985	323.9902	387.5786	432.1122
DDP10	4759	4974	5350	2236.6866	2308.146	2324.749
DDP11	4346	4860	4973	1962.1604	2236.056	2406.352
DDP12	4523	5059	5453	2152.6979	2505.917	2805.742
DDP13	4800	5362	5594	2280.077	2748.891	2956.206
DDP14	4220	4986	5238	1735.3055	2434.829	2559.569
DDP15	4610	4944	5456	2064.664	2489.012	2860.294
DDP16	4781	4959	5189	2315.1469	2422.619	2603.635
DDP17	4271	5754	6064	1728.576	2991.734	3168.29
DDP18	4402	5453	5657	1978.0443	2839.279	2834.144

## Data Availability

Not applicable.

## Abbreviations

The following abbreviations are used in this manuscript:

AVOA	African vulture optimization algorithm
IAVOA	Improved African vulture optimization algorithm
GA	Genetic algorithm
FJSP	The problem of flexible job shop scheduling
JSP	The traditional job shop scheduling problem
DRCFJSP	The dual-resource flexible job shop scheduling problem
